# Integrating telemedicine into dental workflows for medically complex patients: An exploratory pilot feasibility and care coordination study

**DOI:** 10.1177/20552076261490257

**Published:** 2026-09-12

**Authors:** Shawn Adibi, Mahshid Mohajeri, Julian Nathaniel Holland, Elizabeth Scott Hamilton, Lorena Millan, Bonita A. Wynkoop, Chelsea J. Guy-Frank, Mary C. Farach-Carson

**Affiliations:** 1 Department of General Practice and Dental Public Health, School of Dentistry, 67334 The University of Texas Health Science Center at Houston, Houston, TX, USA; 2 Office of Research, School of Dentistry, 67334 The University of Texas Health Science Center at Houston, Houston, TX, USA; 3 Department of Oral and Maxillofacial Surgery, School of Dentistry, 67334 The University of Texas Health Science Center at Houston, Houston, TX, USA; 4 Department of Diagnostics and Biomedical Sciences, School of Dentistry, 67334 The University of Texas Health Science Center at Houston, Houston, TX, USA

**Keywords:** telemedicine, dental care, hypertension, diabetes, oral health assessment, medically complex patients

## Abstract

**Objective:**

This pilot study evaluated the operational feasibility, workflow integration, and care coordination of an on-site telemedicine clearance pathway (the Medically Integrated Oral Health Program) for medically complex dental patients presenting with hypertension and/or diabetes mellitus.

**Methods:**

A non-randomized, two-arm interventional (quasi-experimental) pilot study was conducted with a pragmatic sample of 20 adult dental patients (11 Telemedicine group, 9 Conventional referral group). Allocation was participant selected. Evaluated feasibility metrics included protocol adherence, missed appointments, and data completeness across a 12-month period. Descriptive clinical and laboratory parameters, including Oral Health Risk Assessment Value Index (OHRAVI) scores, body mass index (BMI), blood pressure (BP), and HbA1c, were tracked longitudinally at approximately three-month intervals.

**Results:**

The in-office telemedicine protocol was successfully executed at baseline. However, substantial patient attrition was observed over time, with retention dropping to 54.5% in the Telemedicine group and 44.4% in the Conventional group by the first follow-up. Descriptive observations indicated stable median OHRAVI scores and relative stability in median BMI within the telemedicine cohort, whereas the conventional cohort exhibited slight increases in weight metrics. Blood pressure data demonstrated high variability across both groups. Follow-up HbA1c data were heavily limited by missing data and sparse reporting, which precluded statistical or causal interpretation. Total appointment non-adherence was comparable between groups.

**Conclusion:**

The on-site telemedicine clearance pathway was operationally feasible at initial presentation. However, severe attrition and incomplete longitudinal data prevented reliable evaluation of comparative clinical effectiveness or sustained care coordination. These findings primarily identify implementation and data-collection challenges that should inform a future adequately powered study.

## Plain Language Summary

This small pilot study examined whether an on-site telemedicine pathway could be incorporated into dental care for patients with hypertension and/or diabetes. Eleven participants selected telemedicine and nine selected conventional external referrals. The telemedicine consultation could be completed at the initial visit, but many participants in both groups did not return for follow-up, and clinical data became too incomplete for reliable comparisons. The study therefore provides information about workflow feasibility and implementation challenges, but it cannot establish that telemedicine improved clinical outcomes. A larger study with stronger follow-up procedures is needed. This study was not prospectively registered.

## Introduction

Individuals with multi-systemic conditions such as diabetes mellitus (DM) or hypertension (HTN) are particularly vulnerable to a range of systemic and oral health complications.^1,2^ Both diseases have been associated with altered salivary gland function, periodontal disease, and impaired wound healing, complicating the delivery of dental care and increasing the risk of adverse oral health outcomes.^3^ For example, poorly controlled blood glucose levels can lead to xerostomia, oral infections, and delayed healing following dental procedures, while hypertension and its pharmacological management are linked to dry mouth and gingival enlargement.^4^

Managing dental care in medically complex patients often requires close collaboration between dental and medical providers. Conventionally, dentists refer patients with uncontrolled systemic conditions to their primary care physicians or specialists for evaluation and clearance prior to initiating dental treatment.^5,6^ However, this process can result in significant delays in dental care, missed appointments, or patients abandoning treatment altogether. Delays are further exacerbated by barriers such as health literacy gaps, dental anxiety, and limited access to timely medical appointments.^7^

The COVID-19 pandemic accelerated the adoption of telemedicine and teledentistry as alternatives to traditional in-office care, highlighting their potential to improve care coordination for medically complex patients.^8,9^ While telemedicine can enhance access and expedite medical decision-making in various medical settings, evidence supporting its integration within dental care workflows remains limited.^10,11^ In particular, the practical implications of integrating telemedicine consultations for medical evaluation during dental visits have not been thoroughly explored.

To address this gap, we developed the pilot “Medically Integrated Oral Health Program (MIOHP)” at The University of Texas Health Science Center at Houston School of Dentistry (UTSD), designed to facilitate on-site medical consultations via telemedicine for patients requiring medical evaluation before dental procedures. This prospective, non-randomized pilot evaluation examined operational feasibility, workflow integration, care coordination, follow-up retention, data completeness, and exploratory clinical measures among patients selecting telemedicine or conventional physician referral. We hypothesized that the telemedicine pathway would permit more immediate medical evaluation; all comparisons of clinical measures were exploratory and hypothesis-generating.

## Materials and methods

### Study design and pilot framework

This study was conducted as an exploratory, prospective, non-randomized, two-arm interventional (quasi-experimental) pilot evaluation at the University of Texas Health Science Center at Houston School of Dentistry (UTSD) between January 2020 and December 2023. The primary focus was the operational feasibility, workflow integration, and care coordination of an on-site telemedicine clearance pathway, the Medically Integrated Oral Health Program (MIOHP), compared with conventional external physician referral. Reporting was guided primarily by the TREND statement for non-randomized evaluations, with supplementary consideration of the CONSORT extension for pilot and feasibility trials.

### Participant selection and allocation strategy

A pragmatic target sample size of 20 participants was selected. Because this exploratory pilot evaluated protocol viability and recruitment workflows, no formal a priori power calculation was performed. Participants were screened by trained predoctoral providers in the UTSD Assessment Clinic under faculty supervision.

Eligible participants were adults aged 18 years or older with a documented medical history or self-report of type 2 diabetes mellitus and/or hypertension requiring medical evaluation or clearance before invasive dental treatment. Exclusion criteria were absence of a qualifying systemic condition, age younger than 18 years, or unwillingness to provide written informed consent.

A total of 56 patients were assessed for eligibility. Thirty-six withdrew consent before allocation, leaving 20 participants for enrollment. Allocation was non-randomized and based on participant preference: 11 participants selected the Telemedicine pathway and 9 selected the Conventional referral pathway. All participants received their selected initial pathway. Because of the nature of the intervention and participant-selected allocation, participants and treating clinicians could not be blinded to the selected pathway. Outcome assessors and the statistical analyst were also not blinded to group allocation.

### Study arms and operational protocols

*Telemedicine Workflow (MIOHP):* Participants choosing the telemedicine arm utilized a full-service digital health partner, Sesame Care. Telehealth consultations were conducted immediately via an app-based interface or FaceTime on mobile devices within a designated private room at UTSD. Grant funding from the Dental Trade Alliance Foundation covered the cost of the initial consultation.

During the appointment, clinic staff recorded point-of-care clinical metrics, including BP and a fingerstick blood glucose test. These values were transmitted directly to the remote, licensed Telehealth Provider. Long-term glycemic management was assessed via HbA1c testing through an affiliated outside laboratory. Remote medical providers managed the patient’s condition and issued clinical decisions or adjustments based on the ACC/AHA Hypertension Guidelines^12^ and the American Diabetes Association Standards of Care.^13^ Upon medical clearance, the provider returned a signed digital assessment to the dental clinic.

*Conventional Referral Pathway:* Participants in the Conventional group were provided a standardized UTSD paper referral form detailing their in-office vitals and clinical requirements. They were instructed to schedule and attend an appointment with their external primary care physician or specialist to obtain medical clearance. These patients were discharged from the assessment clinic without receiving further dental treatment until physical documentation of medical clearance was returned to UTSD.

### Longitudinal follow-up and exploratory measures

After documented medical clearance through either pathway, participants were scheduled for longitudinal monitoring for up to 12 months at approximately three-month intervals. At baseline and each available follow-up visit, study personnel collected exploratory measures to assess retention, data completeness, and clinical trends. Adverse events and unintended effects associated with either care pathway were monitored throughout the study period.

*Care Coordination and Feasibility Metrics:* Protocol adherence was summarized using retention at each time point and total missed appointments recorded in the axiUm electronic health record. Oral Health Status: The Oral Health Risk Assessment Value Index (OHRAVI) was recorded on a scale of 0–1 (minor/good), 1–2 (moderate complexity), and 2–3 (complex/poor condition). Systemic Health Parameters: Body mass index (BMI, kg/m^2^), systolic and diastolic blood pressure (mmHg), and available longitudinal HbA1c results requested from treating physicians were recorded.

### Statistical analysis

The individual participant was the unit of analysis. Descriptive statistics were used primarily to evaluate feasibility, retention, data completeness, and observed clinical trajectories. Continuous variables were summarized as medians and interquartile ranges, and categorical variables as frequencies and percentages. Because of the small sample and non-normal distributions, exploratory between-group comparisons at individual time points used Mann-Whitney U tests for continuous variables and Fisher’s exact tests for categorical variables. Analyses were performed using available observed data at each time point; participants with missing measurements were excluded only from the corresponding outcome analysis, and no missing values were imputed. Accordingly, denominators varied by outcome and time point. Analyses were performed with IBM SPSS Statistics, version 27. P values were considered exploratory; no adjustment for multiple testing, baseline confounding, or participant self-selection was performed. The analyses were not intended to support causal inference.

### Ethical considerations

This study did not alter the standard dental care provided to participants. Participants remained responsible for routine medical testing and follow-up outside UTSD. The study covered the initial telemedicine consultation for participants in the Telemedicine group. Written informed consent was obtained from all participants. The study complied with the Declaration of Helsinki and Health Insurance Portability and Accountability Act requirements and was approved by the Committee for the Protection of Human Subjects at UTHealth Houston (protocol number HSC-DB-20-0029).

## Results

**Figure 1.**
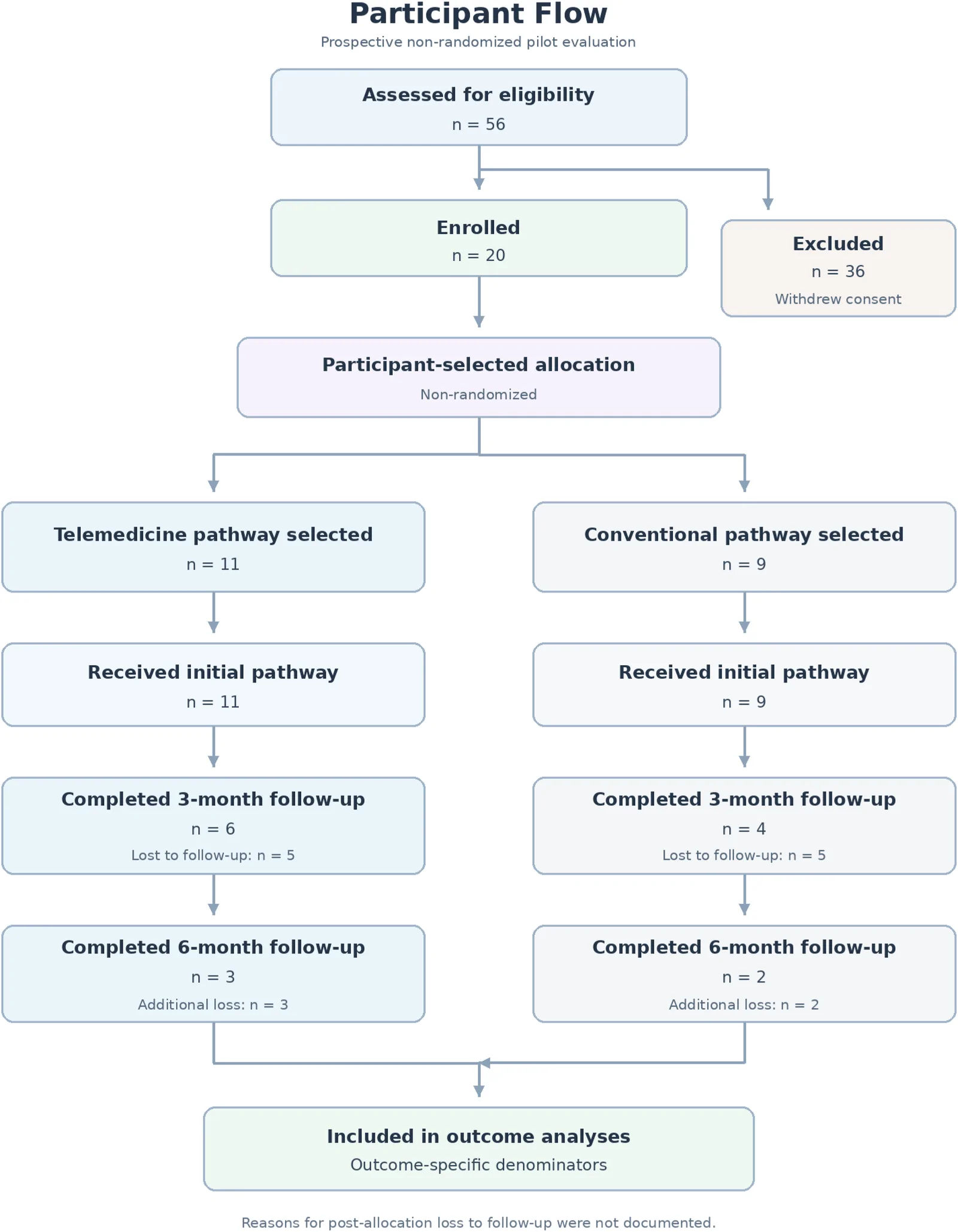
Participant flow through the study. Of 56 patients assessed, 36 withdrew consent before allocation and 20 enrolled. By participant preference, 11 selected and received telemedicine and 9 selected and received conventional referral. At 3 and 6 months, 6 and 3 telemedicine participants and 4 and 2 conventional participants completed follow-up, respectively. Reasons for post-allocation loss were not documented; outcome-specific denominators are reported in Table 2.

Of the 56 patients assessed for eligibility, 36 withdrew consent before allocation, and 20 were enrolled in the study. Eleven participants selected and received the Telemedicine pathway, while nine selected and received the Conventional referral pathway. All 20 participants completed the initial pathway assessment. At the 3-month follow-up, 6 of 11 participants in the Telemedicine group and 4 of 9 in the Conventional group remained under evaluation. At 6 months, the corresponding numbers were 3 of 11 and 2 of 9, respectively. Reasons for loss to follow-up after pathway allocation were not documented (Figure 1).

### Participant characteristics

Twenty participants were enrolled: 11 selected the Telemedicine pathway and 9 selected conventional referral. Baseline demographic and clinical characteristics are reported in Table 1. Median age was 66 years (IQR 52.0–75.0) in the Telemedicine group and 58 years (IQR 47.5–68.5) in the Conventional group. Hypertension was the most prevalent condition; 36.4% and 44.4% of participants, respectively, had both hypertension and diabetes. Exploratory tests did not detect statistically significant baseline differences in weight, BMI, blood pressure, or OHRAVI scores. However, with this small self-selected sample, absence of statistical significance should not be interpreted as evidence that the groups were equivalent.

**Table 1.** Baseline demographic and clinical characteristics.

Characteristic	Telemedicine group (n=11)	Conventional group (n=9)
Age, (Median (IQR))	66.0 (52.0 - 75.0)	58.0 (47.5 - 68.5)
Gender, n (%)		
Male	6 (54.5%)	4 (44.4%)
Female	5 (45.5%)	5 (55.6%)
Race, n (%)	​	​
White	6 (54.5%)	1 (11.1%)
Hispanic	2 (18.2%)	6 (66.7%)
African American	3 (27.3%)	2 (22.2%)
Underlying Disease, n (%)		
Hypertension only	6 (54.5%)	4 (44.4%)
Diabetes only	0 (0%)	0 (0%)
Hypertension & Diabetes	4 (36.4%)	4 (44.4%)
Vaping, n (%)		
Yes	1 (100%)	0 (0%)
No	0 (0%)	9 (100%)
Tobacco Use, n (%)		
Yes	2 (18.2%)	1 (11.1%)
No	8 (72.7%)	8 (88.9%)
Cannabis	1 (9.1%)	0 (0%)
Insurance, n (%)		
Private	4 (36.4%)	1 (11.1%)
Public	1 (9.1%)	3 (33.3%)
None	2 (18.2%)	4 (44.4%)

### Feasibility metrics: Longitudinal retention and data completeness

A primary outcome of this pilot framework was data completeness and protocol adherence over time. Baseline attendance was 100% in both pathways. At 3 months, 6 of 11 participants (54.5%) in the Telemedicine group and 4 of 9 (44.4%) in the Conventional group completed follow-up. At 6 months, the corresponding numbers were 3 of 11 (27.3%) and 2 of 9 (22.2%) (Figure 1). These are overall visit-retention counts. Clinical outcome data were available for 5 Telemedicine and 4 Conventional participants at 3 months and for 3 and 2 participants, respectively, at 6 months (Table 2). Reasons for post-allocation loss to follow-up were not documented.

**Table 2.** Clinical measures at baseline, follow-up 1, and follow-up 2.

​	Baseline		Follow-up 1 (3 months)		Follow-up 2 (6 months)	
Variable*	Telemedicine (n=11)	Conventional (n=9)	Telemedicine (n=5)	Conventional (n=4)	Telemedicine (n=3)	Conventional (n=2)
Weight (lbs)	200.0 (160.5-216.0)	200.0 (191.0-211.0)	170.5 (153.75-194.0)	227.5 (193.75-255.0)	200.0 (185.0-230.0)	234.0 (219.5-248.5)
BMI (kg/m^2^)	27.9 (27.6-33.1)	32.8 (27.1-34.2)	27.75 (27.45-27.9)	33.5 (30.4-34.575)	27.9 (27.25-33.95)	34.4 (33.75-35.05)
OHRAVI Score	1.2 (0.9-1.6)	1.0 (0.6-1.3)	1.25 (1.2-1.525)	0.9 (0.45-1.35)	1.2 (0.66-1.2)	0.8 (0.55-1.05)
Systolic BP (mmHg)	155.0 (138.5-172.5)	144.0 (115.0-165.0)	143.0 (131.0-146.0)	121.0 (112.5-131.0)	123.0 (111.0-131.5)	118.5 (110.25-126.75)
Diastolic BP (mmHg)	83.0 (79.5-88.0)	79.0 (74.0-89.0)	76.0 (70.0-85.75)	65.0 (62.5-69.0)	68.0 (66.0-73.0)	68.5 (67.75-69.25)
HbA1c	5.95 (5.925-5.975)	7.4 (5.8-7.7)	6.0 (6.0-6.0)	6.05 (5.475-6.625)	N/A	5.5 (5.5-5.5)

*Values are median (IQR). Column denominators are outcome-data denominators for weight, BMI, OHRAVI, and blood pressure. HbA1c denominators were Telemedicine/Conventional n=3/2 at baseline, n=1/2 at Follow-up 1, and n=0/1 at Follow-up 2. No missing values were imputed.

**Figure 2.**
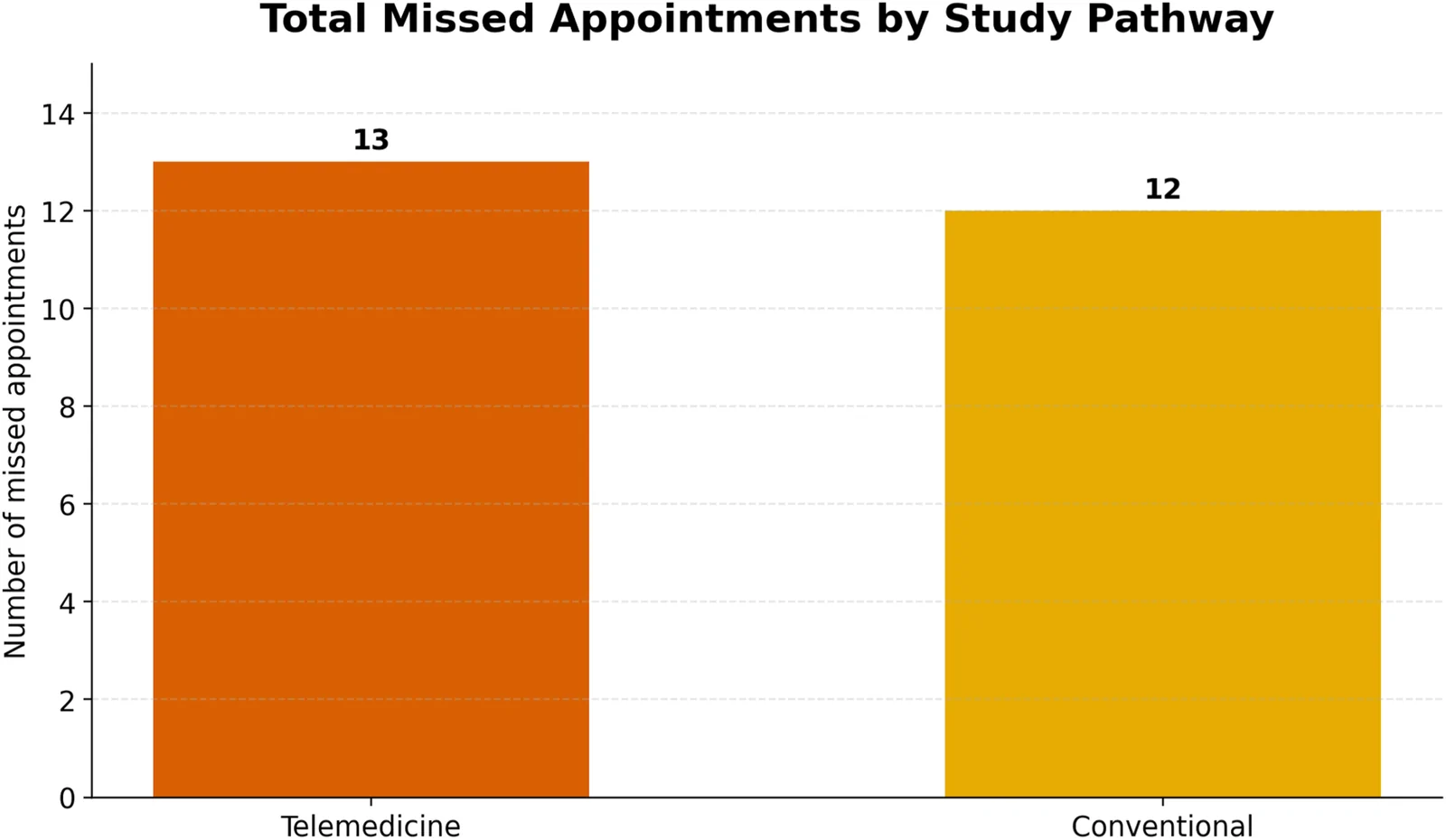
Total missed appointments by study pathway. Thirteen missed appointments were recorded in the Telemedicine group and 12 in the Conventional group over the full study period. These are appointment counts, not numbers of unique participants.

Total missed appointments over the study period were similar: 13 were recorded in the Telemedicine group and 12 in the Conventional group (Figure 2). These values represent appointment counts rather than numbers of unique participants.

### Dental case completion

Time from enrollment to dental case completion was intended as a care-coordination metric, but high attrition and incomplete workflow records prevented robust estimation for the full cohort. In the Telemedicine group, the initial on-site consultation allowed medical evaluation and, when clearance was granted, initiation of dental planning on the same day. Participants in the Conventional group were required to obtain external medical evaluation before clearance. Long-term comparison of case completion between pathways was not possible because of missing follow-up data.

### Oral health trends (OHRAVI)

**Figure 3.**
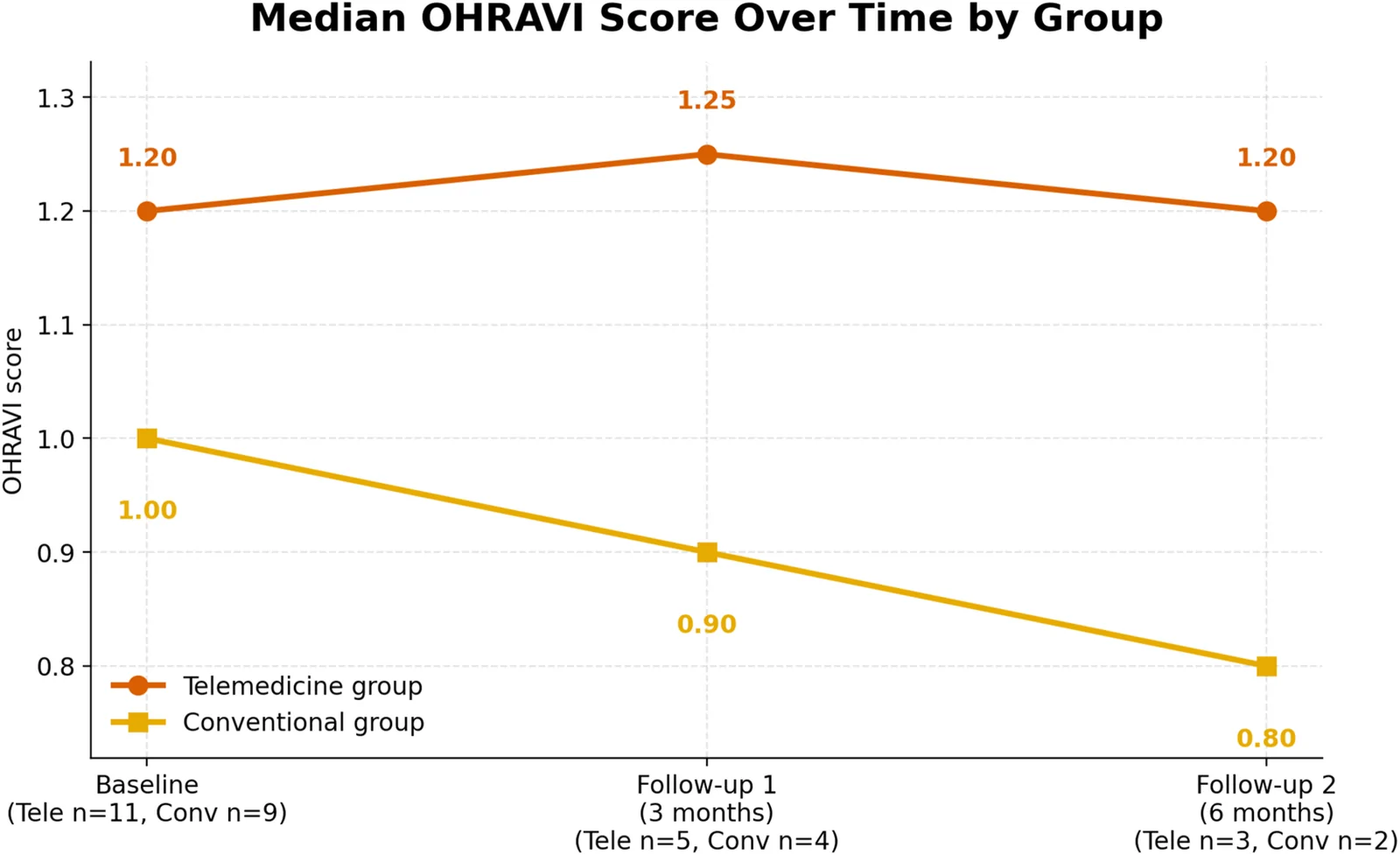
Median Oral Health Risk Assessment Value Index (OHRAVI) scores over time. Values are shown for the Telemedicine and Conventional groups at baseline, 3 months, and 6 months. Outcome-specific denominators were Telemedicine/Conventional n=11/9, 5/4, and 3/2, respectively. Values are descriptive because of participant-selected allocation and substantial attrition.

At baseline, median OHRAVI scores were 1.2 (IQR 0.9–1.6) in the Telemedicine group and 1.0 (IQR 0.6–1.3) in the Conventional group. At the first follow-up, the corresponding medians were 1.25 (IQR 1.2–1.525; n=5) and 0.9 (IQR 0.45–1.35; n=4). At the second follow-up, the medians were 1.2 (IQR 0.66–1.2; n=3) and 0.8 (IQR 0.55–1.05; n=2), respectively. These observations are descriptive and should not be interpreted as evidence of a between-group effect (Figure 3; Table 2).

### Anthropometric and metabolic tracking (weight, BMI, and HbA1c)

**Figure 4.**
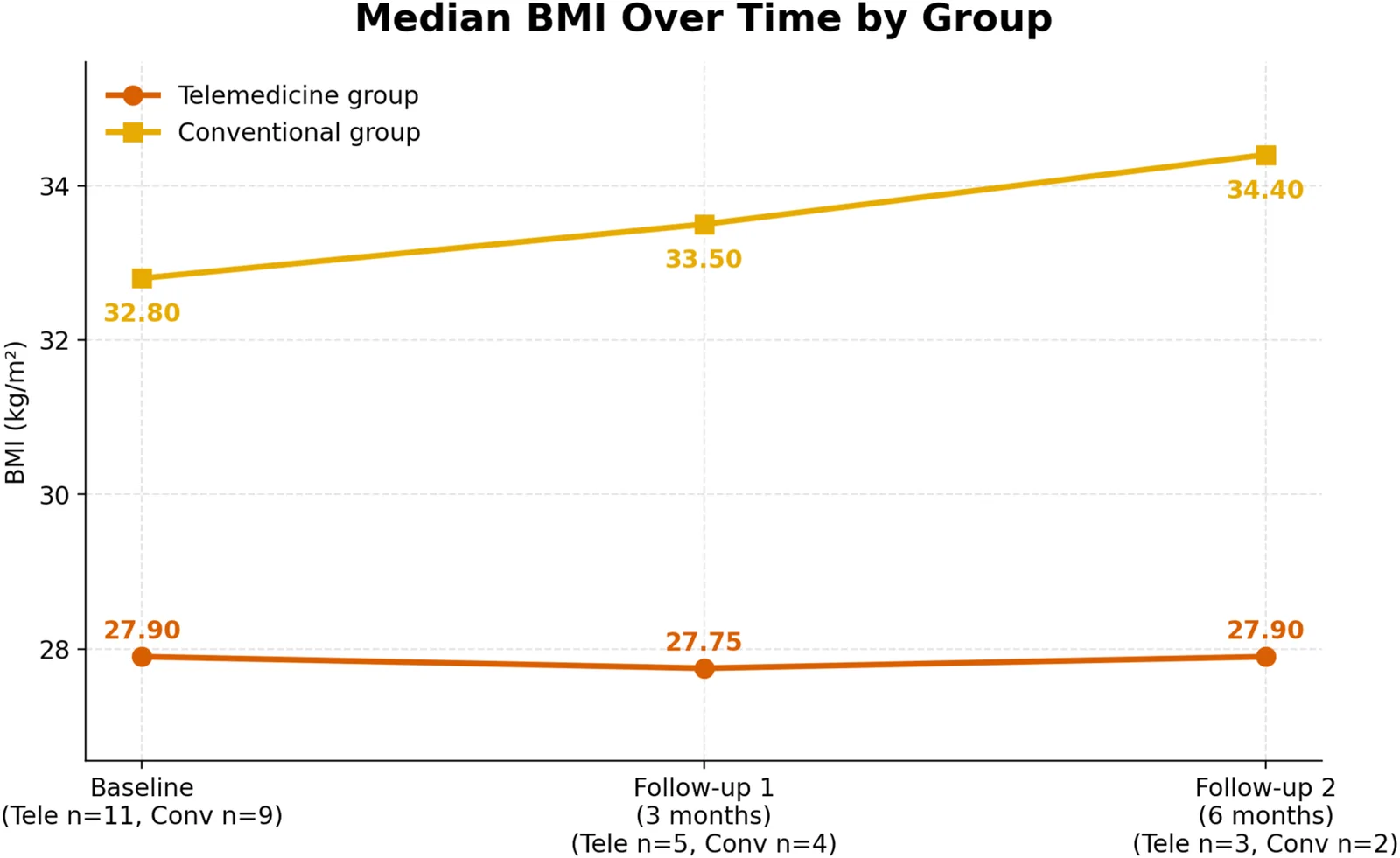
Median body mass index (BMI) over time. Values are shown for the Telemedicine and Conventional groups at baseline, 3 months, and 6 months. Outcome-specific denominators were Telemedicine/Conventional n=11/9, 5/4, and 3/2, respectively. Later estimates reflect small, changing subsets and should not be interpreted as intervention effects.

Median baseline weight was 200.0 lb in both groups. Among the small and changing subsets with follow-up data, observed median weights differed at later time points. Exploratory Mann–Whitney tests produced low p values at the first and second follow-ups; however, these estimates are highly vulnerable to selection bias, baseline imbalance among retained participants, attrition, missing data, and multiple testing. They therefore do not demonstrate a treatment effect. BMI patterns were similarly unstable (Figure 4).

Laboratory reporting for HbA1c was extremely sparse. Baseline values were successfully captured for only five participants (three Telemedicine, two Conventional). Follow-up data were available for three participants at the first follow-up (Telemedicine n=1; Conventional n=2) and one participant at the second follow-up (Telemedicine n=0; Conventional n=1). No formal statistical or causal comparisons could be conducted on glycemic data due to these missing values.

### Hemodynamic monitoring (blood pressure)

**Figure 5.**
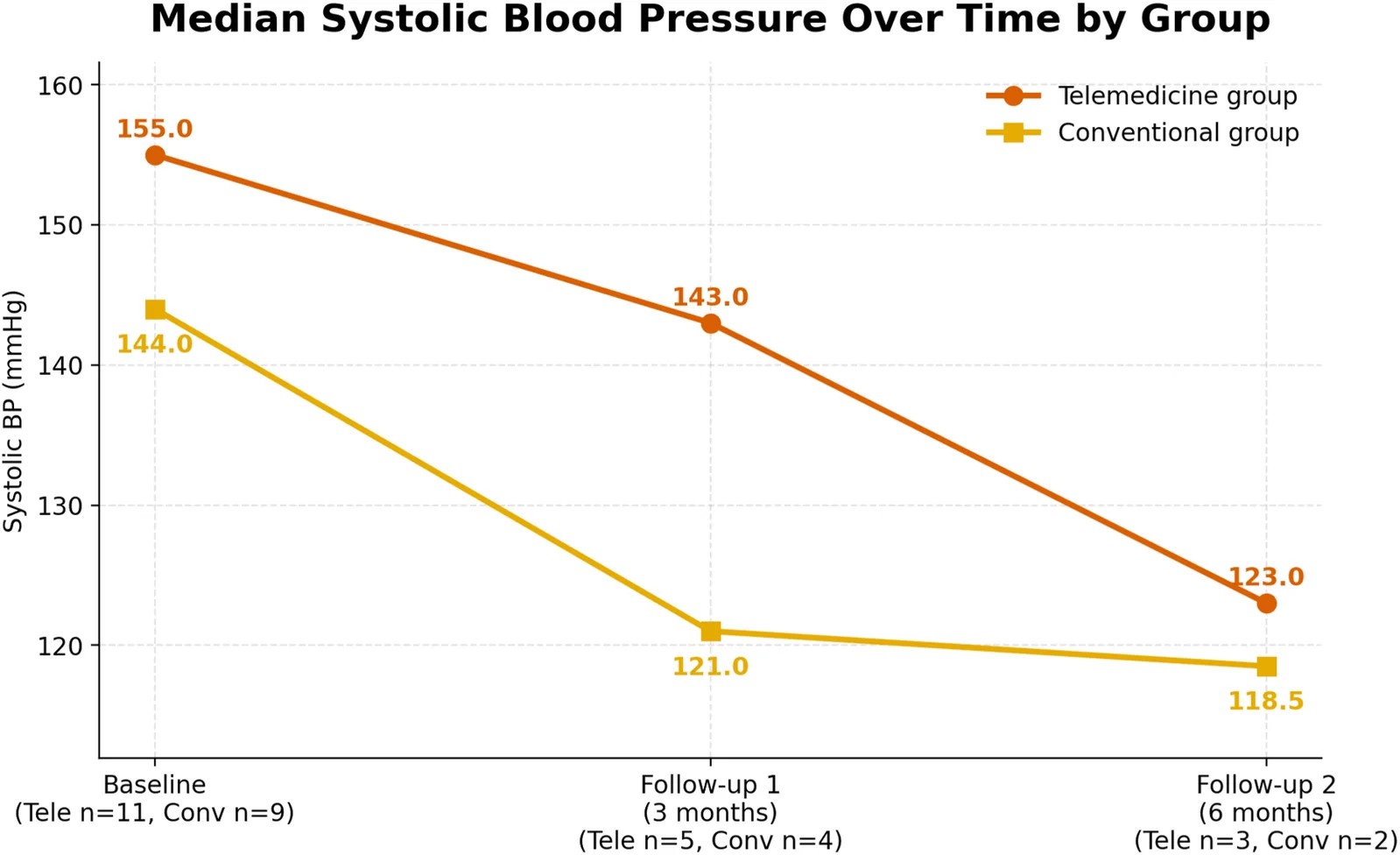
Median systolic blood pressure over time. Values are shown for the Telemedicine and Conventional groups at baseline, 3 months, and 6 months. Outcome-specific denominators were Telemedicine/Conventional n=11/9, 5/4, and 3/2, respectively. Values correspond to Table 2 and are descriptive.

**Figure 6.**
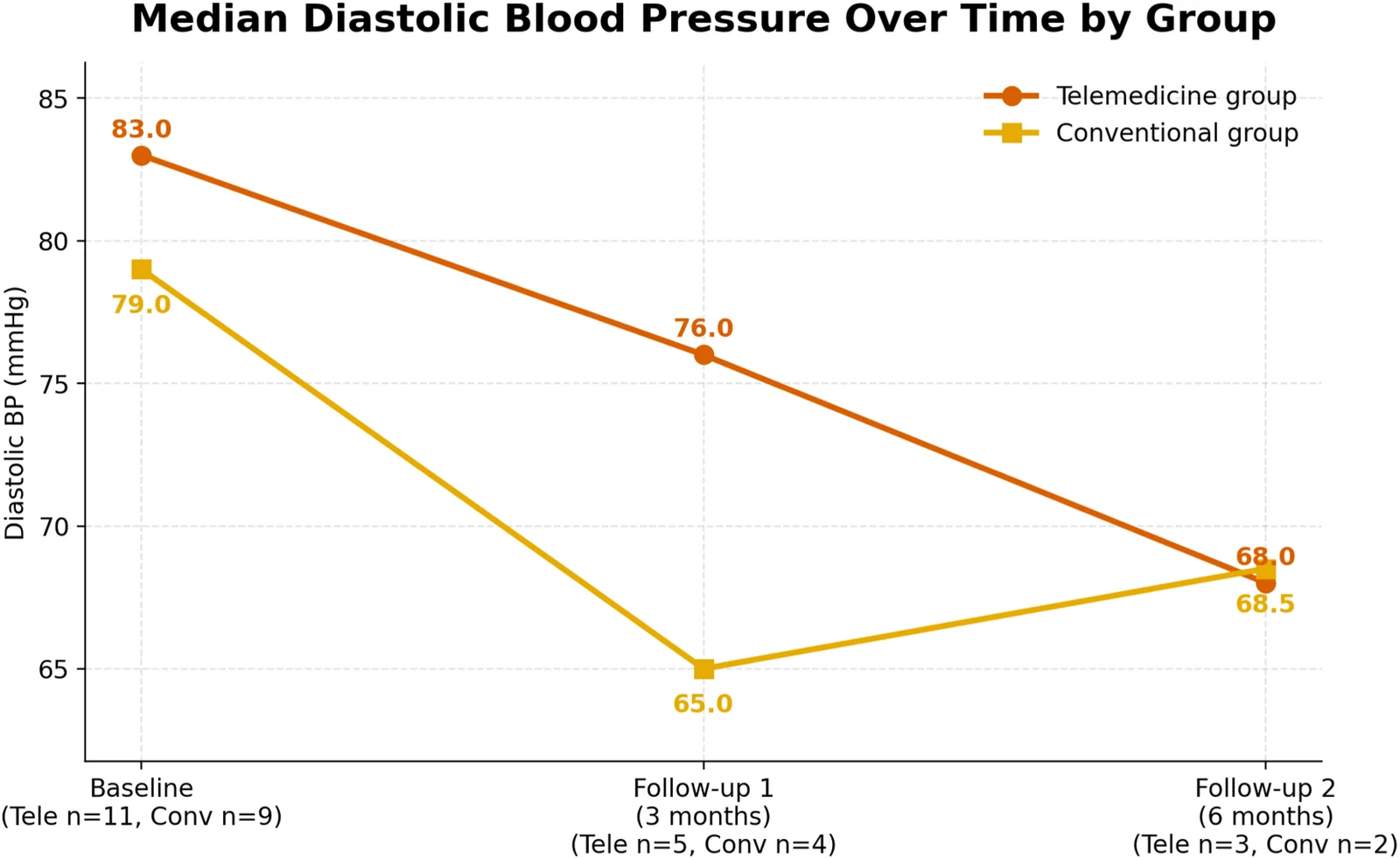
Median diastolic blood pressure over time. Values are shown for the Telemedicine and Conventional groups at baseline, 3 months, and 6 months. Outcome-specific denominators were Telemedicine/Conventional n=11/9, 5/4, and 3/2, respectively. Values correspond to Table 2 and are descriptive.

Baseline median systolic blood pressure was 155.0 mmHg (IQR 138.5–172.5) in the Telemedicine group and 144.0 mmHg (IQR 115.0–165.0) in the Conventional group. At the first follow-up, the corresponding medians were 143.0 and 121.0 mmHg; at the second follow-up, they were 123.0 and 118.5 mmHg. Diastolic blood pressure showed similarly variable descriptive patterns. Figures 5 and 6 and Table 2 present the outcome-specific values and denominators. No reliable between-group inference regarding blood-pressure control can be made.

### Adverse events and unintended effects

No intervention-related adverse events or unintended harmful effects were reported in either study group during the study period.

## Discussion

This pilot study shows that an on-site telemedicine consultation could be implemented at the initial dental visit for medically complex patients. Its principal contribution is information about operational feasibility and the substantial difficulty of maintaining longitudinal follow-up. Because allocation was participant-selected, the sample was very small, attrition was severe, and outcome data were incomplete, the study cannot determine whether telemedicine improved oral health, BMI, glycemic control, blood pressure, appointment adherence, or long-term dental case completion.

The ability to conduct an immediate telemedicine consultation is consistent with the proposed role of teledentistry in facilitating access and coordination when in-person medical evaluation is difficult.^14^ However, feasibility of delivering an initial consultation should be distinguished from effectiveness. The present data support initial workflow implementation but do not establish improvement in clinical outcomes or sustained engagement.

Observed OHRAVI values were broadly similar between pathways, but the number of participants contributing follow-up data rapidly declined. Although previous studies have described potential benefits of telehealth for preventive oral-health services,^15^ the current study cannot attribute the observed OHRAVI patterns to telemedicine because of self-selection, attrition, and the absence of adjustment for confounding.

Blood-pressure values were highly variable in both groups. The observed distributions do not support conclusions about greater personalization, treatment adjustment, or superior stability in either pathway. Comparisons with previous telemedicine studies^16^ should therefore be interpreted as contextual rather than confirmatory evidence.

HbA1c data were available for only five participants at baseline and became even sparser during follow-up. Consequently, no conclusion can be drawn regarding glycemic monitoring or control. Any apparent pattern may reflect missingness and the characteristics of participants who remained under observation rather than an intervention effect.

Similarly, later differences in weight and BMI were derived from very small, changing subsets of participants. Although telemedicine may theoretically support engagement and health behavior,^17^ this mechanism was not directly measured, and the present results do not demonstrate improved weight management.

## Implications for healthcare delivery

The findings support the practicality of offering an initial on-site telemedicine consultation within a dental clinic. This pathway may reduce the immediate logistical step of arranging an external consultation, but the study did not provide sufficiently complete data to quantify effects on treatment delay, case completion, missed appointments, provider burden, or clinical outcomes.

Potential applications in underserved populations remain plausible but were not directly evaluated. Wider implementation would require reliable connectivity, suitable devices, privacy safeguards, technical support, standardized clearance procedures, and deliberate strategies for participant retention and longitudinal data capture.

A future study should prospectively define feasibility outcomes and progression criteria, use randomization or a robust adjustment strategy where feasible, document participant flow and reasons for attrition, standardize follow-up intervals and outcome measurement, and prespecify methods for handling missing data and confounding.

## Limitations

This study has several important limitations. The sample of 20 participants was pragmatic and not statistically powered for comparative effectiveness. Estimates are imprecise, and hypothesis tests at later time points are unstable because very few participants remained under observation.

Participant-selected allocation creates a high risk of selection bias and confounding. Preferences, technology access, motivation, disease severity, access to primary care, and other measured or unmeasured characteristics may have influenced both pathway selection and outcomes. No matching, multivariable adjustment, propensity-based method, or other confounding-control strategy was used.

Attrition was severe in both groups and produced substantial, potentially informative missingness. The analyses therefore reflect changing subsets rather than comparable longitudinal cohorts. Reasons for loss to follow-up were not fully documented in the available dataset, and the study could not determine whether participants who remained differed systematically from those lost.

Technical problems, including unstable internet connections and device-compatibility issues, occasionally disrupted telemedicine visits. Intervention fidelity, consultation duration, provider-level variation, participant experience, and adverse events were not systematically quantified in the available report.

The single-center setting limits generalizability. The planned 12-month follow-up was substantially compromised by attrition, and the available data cannot establish durability, comparative clinical effectiveness, or causal mechanisms. These findings should be interpreted as feasibility observations and should not guide clinical policy without confirmation in a larger, prospectively designed study.

## Conclusions

This exploratory pilot demonstrates that an on-site telemedicine medical-clearance consultation can be initiated within a dental-clinic workflow. It also identifies major barriers to longitudinal evaluation, particularly participant attrition and incomplete outcome data.

The study does not establish superiority of telemedicine over conventional referral for clinical outcomes, adherence, or long-term case completion. Its findings are descriptive and hypothesis-generating because of participant-selected allocation, small sample size, confounding, and severe missingness.

Future research should use a prospectively registered protocol, explicit progression criteria, stronger recruitment and retention procedures, standardized outcome collection, and an adequately powered randomized or rigorously adjusted comparative design.

## Data Availability

The data supporting this study are available from the corresponding author upon reasonable request, subject to ethical and legal considerations.*
